# Perception of Non-Alcoholic Fatty Liver Disease: Real-Life Experience From Pakistan

**DOI:** 10.7759/cureus.16029

**Published:** 2021-06-29

**Authors:** Nazish Butt, Muhammad Ali Khan, Lajpat Rai, Riaz Hussain Channa, Hanisha Khemani, Amanullah Abbasi

**Affiliations:** 1 Gastroenterology, National Medical Centre, Karachi, PAK; 2 Gastroenterology, Aga Khan University Hospital, Karachi, PAK; 3 Gastroenterology, Jinnah Postgraduate Medical Centre, Karachi, PAK; 4 Internal Medicine, Jinnah Postgraduate Medical Centre, Karachi, PAK; 5 Gastroentrology, Jinnah Postgraduate Medical Centre, Karachi, PAK; 6 Internal Medicine, Dow University of Health Sciences, Karachi, PAK

**Keywords:** nonalcoholic fatty liver disease (nafld), fatty liver disease, patients’ perception, karachi, pakistan

## Abstract

Introduction

Non-alcoholic fatty liver disease (NALFD) has become one of the most pervasive causes of hepatic pathology. Because of its marked association with metabolic syndrome, type II diabetes and cardiovascular disease, NAFLD has gained substantial focus recently. Its prevalence and incidence are on the rise in Pakistan. However, due to its indolent and mostly asymptomatic course, NAFLD is often overlooked. This reckless behavior towards a potentially deadly disease is influenced most notably by disinformation or flawed perception, although there are a number of other complex socioeconomic components to this as well. With respect to NAFLD, the gap between disease understanding and steps for management is growing in the Pakistani society. With this study, we hoped and aimed to evaluate just how far and wide these shortcomings were found and how was NAFLD perceived in the local populace via a self-administered survey.

Methods

This was a cross-sectional observational cohort study undertaken at the Department of Gastroenterology, Jinnah Postgraduate Medical Centre, and Medical Unit II, Dow University of Health Sciences, Ojha Campus, Karachi, Pakistan. All patients ≥18 years with a diagnosis of NAFLD were included in the study. NAFLD was diagnosed on the basis of sonographic evidence. All ultrasounds were done by a senior expert radiologist with at least 10 years of postgraduate experience. Ultrasounds were performed twice in all patients to rule out human error and bias. Perceptions regarding the knowledge of NAFLD were assessed using a self-administered survey questionnaire.

Results

The female-to-male ratio in our cohort was 3:1. The mean age and body mass index (BMI) recorded were 39.85 ± 9.79 years and 31.21 ± 3.6 kg/m^2^, respectively. Sixty participants (26.4%) knew about their disease (NAFLD) while only 36 (15.9%) knew what NAFLD was and only 33 (14.5%) participants knew about the cardiovascular risk associated with it. Nearly two-thirds of the patients considered themselves overweight, while 180 (79.3%) of them were willing to lose weight. However, just about half of the cohort admitted the need for improved eating habits and increased physical activity/exercise in their daily lives. Fifty-seven (25.1%) patients admitted to using alternative or quack medications and only 45 (19.8%) patients considered them harmful.

Conclusions

Patients harboring NAFLD have little to no knowledge about the disease and its nature or the fact that they are suffering from it despite being diagnosed clinically. Furthermore, while the general populace is willing to accept being overweight and having unhealthy eating habits, their willingness in initiating real-life practical steps to manage NAFLD is lacking.

## Introduction

Ludwig et al. first described an unnamed liver disease in 1980 that imitated alcoholic hepatitis and could lead to cirrhosis [1]. This disease was characterized by the accumulation of abnormal levels of fat within the hepatic parenchyma, evident by radiological or histological examination and occurring in the absence of other causes of liver injury, such as alcoholism, chronic hepatitis, inherited metabolic liver disorders, and drugs. Further examination of biopsy specimens revealed lobular hepatitis, focal necrosis with mixed inflammatory infiltrates, Mallory bodies, and even cirrhosis. This non-alcoholic hepatic disorder would eventually be known as non-alcoholic fatty liver disease (NAFLD) [2].

In the few decades since its first report, NALFD has become one of the most pervasive causes of hepatic pathology. With the currently declining incidence of chronic viral hepatitis and a burgeoning obesity epidemic, NAFLD will become the leading cause of liver disease universally sooner than later [3]. The approximate prevalence of NAFLD worldwide is 25%, putting the burden of this disease at an astonishing 1.7 billion patients [4]. Higher prevalence rates have been recorded in the Middle Eastern nations and the USA, whereas much lower prevalence rates are reported from Africa [4]. Nonetheless, NAFLD is now becoming endemic in every nation and continent on the planet and accounts for a major share of healthcare costs with each passing year [5].

Perception is reality. The general populace can only be expected to follow protocols, implement healthy nutritional regimens, make necessary adjustments to their daily routines, avoid precipitating factors, and adapt to the changing dynamics of a disease if they perceive that disease to be a threat to their own well-being [6-9]. The current coronavirus disease 2019 (COVID-19) pandemic has made clear the counterintuitive impact of redundant and unscientific attitudes or beliefs in imposing necessary standard operating procedures and vaccination drives for controlling a devastating disease [10,11]. Most, if not all, of these misplaced notions are due to misapprehensions about the disease, its pathophysiology, and eventual outcomes [12,13]. Incorrect perceptions about a disease can lead to irresponsible behaviors, with detrimental actions. For example, in the case of COVID-19, misguided perceptions have led to a calamitous situation in some regions of the world. However, this can occur with any disease process anytime and anywhere, and NAFLD is no exception.

Due to the indolent nature of NAFLD and its largely asymptomatic presentation, patients often misinterpret or misperceive the danger it represents overtime. NAFLD is closely linked to metabolic syndrome (metS) and type II diabetes mellitus [14,15]. Consequently, over half of the mortality in patients with NAFLD occurs because of adverse cardiovascular events [16,17]. The next two major causes of NAFLD morbidity and mortality are cirrhosis and the development of hepatocellular carcinoma (HCC) [18]. These grave outcomes highlight the need for a proper understanding of the disease on the patient’s side and place an equal onus on the physician to recognize the need for monitoring and intervention in patients with NAFLD [19].

Previous studies that have evaluated the perception of NALFD in the general populace have demonstrated an overall indifferent attitude toward the disease, as the participants were either uneducated about the disease or did not consider themselves at risk [20,21]. Other reports exposed clear-cut communication gaps between the patients and physicians with regard to NALFD, as most doctors did not discuss the disease adequately with their subjects and the patients themselves seldom inquired about the disease they were harboring [22]. Most data on patient perceptions of NAFLD are derived from studies conducted in developed nations with high literacy rates, easy access to information, and standardized healthcare systems. A safe assumption is that the same shortcomings would be encountered in the developing world, only more pronounced. In the present study, we surveyed the factors that shape the perception of NAFLD in the Pakistani populace.

## Materials and methods

This cross-sectional observational cohort study was conducted from January 2017 to December 2020 (four years) at the Department of Gastroenterology, Jinnah Postgraduate Medical Centre (JPMC), and Medical Unit II, Dow University of Health Sciences, Ojha Campus, Karachi, Pakistan. Patients aged 18 years or older of either gender who were diagnosed with NAFLD were included in the study. NAFLD was diagnosed via ultrasound carried out twice in all patients to reduce human error and bias. All ultrasound/sonographic examinations were carried out by a member of the team of senior radiologists at either institute, all of whom have postgraduate qualifications and at least 10 years of experience. The spectrum of NAFLD consists of two entities: non-alcoholic fatty liver (NAFL) and non-alcoholic steatohepatitis (NASH). However, for the purposes of this study, we did not distinguish between the two and simply enrolled all patients with fatty infiltration evident on ultrasound. Body mass index (BMI) was calculated by dividing the weight of the patient in kilograms by the height in meters squared.

Patients with chronic viral hepatitis, alcoholic hepatitis, compensated liver disease, renal failure, heart failure, thyroid and parathyroid disorders, terminal illnesses, and metastatic malignancy were all excluded from the study. All biochemical and radiological workups to rule out concomitant diseases were done free of cost to the patients. The institutional review board of JPMC gave approval for this research beforehand. Written consent was obtained in all cases, and patient confidentiality was ensured at all times. Participants were required to fill out a simple questionnaire designed to record the baseline patient characteristics and their perceptions of NAFLD (Table 1). The 12 questions were designed to focus on the main aspects of the disease that may impact eventual outcomes, including knowledge of having the disease and its association with adverse cardiovascular events, the weight of the patients and their physical activity/exercise, dietary habits, and use of quack medications. Questionnaires were always completed in the outpatient department (OPD) in the presence of an attendant. A resident or senior registrar was readily available to address any queries regarding the questionnaire.

**Table 1:** Questionnaire assessing the perception of NAFLD NAFLD = non-alcoholic fatty liver disease; CVD = cardiovascular disease

Please answer the questions as only “Yes” or “No”		
Do you know what disease you have?	Yes	No
Do you know what fatty liver is?	Yes	No
Do you know NAFLD is associated with CVDs?	Yes	No
Do you think you are overweight?	Yes	No
Has anyone told you that you are overweight?	Yes	No
Do you think you need to lose weight?	Yes	No
Do you think you overeat?	Yes	No
Do you think you eat inappropriate food?	Yes	No
Do you think you get enough exercise?	Yes	No
Do you think the chores of the house is exercise?	Yes	No
Have you ever taken Hakeemi medication?	Yes	No
Do you consider Hakeemi medications harmful?	Yes	No

Data were entered and analysed using the Statistical Package for the Social Sciences (SPSS) for Windows, Version 21.0 (IBM Corp., Armonk, NY). A consecutive and non-probability sampling technique was used for this cohort. Means and standard deviations were calculated for the quantitative variables of age and BMI. Frequencies and percentages were computed for the qualitative variables of gender, marital status, residence (urban or rural), and socioeconomic status, and for the responses of either “Yes” or “No” in the questionnaire.

## Results

In total, 227 patients were enrolled in the study. The female-to-male ratio in our cohort was 3:1. The mean age and BMI were 39.85 ± 9.79 years and 31.21 ± 3.6 kg/m^2^, respectively. As expected, a majority of the patients were married and middle aged. The patients were predominantly from urban areas and belonged to the low-income socioeconomic group. The general characteristics of the patients included in the study are summarized in Table 2.

**Table 2:** General characteristics of the patients enrolled in the study

Data	N=227
Age (mean)	39.85 ± 9.79 years
Gender	
Male	57 (25.1%)
Female	170 (74.9%)
Marital status	
Single	27 (11.9%)
Married	193 (85.0%)
Widowed	7 (3.1%)
Residence	
Urban	155 (68.28%)
Rural	72 (31.71%)
Socioeconomic status	
Lower class	153 (67.4%)
Middle class	74 (32.59%)
Body mass index (kg/m^2^)	31.21 ± 3.6

Only 60 (26.4%) patients knew they had NAFLD, and even fewer knew the exact nature of their disease. Most concerning, only 33 (14.5%) of the patients had some idea about the adverse cardiovascular events associated with NAFLD. However, the vast majority of the patients recognized the need for better weight control and admitted to their unhealthy eating habits. Despite this, only 74 (32.6%) recognized the need for more physical activity/exercise in their daily lives. Only a small percentage of the patients had used quack medications; however, most did not view these medications as harmful (see Discussion). The results of the survey are shown in Table 3.

**Table 3:** Results of the survey on the perception of NAFLD NAFLD = non-alcoholic fatty liver disease; CVD = cardiovascular disease

Questions	N=227	
	Yes (%)	No (%)
Do you know what disease you have?	60 (26.4%)	167 (73.6%)
Do you know what NAFLD is?	36 (15.9%)	191 (84.1%)
Do you know NAFLD is associated with CVDs?	33 (14.5%)	194 (85.5%)
Do you think you are overweight?	143 (63.0%)	84 (37.0%)
Has anyone told you that you are overweight?	158 (69.6%)	69 (30.4%)
Do you think you need to lose weight?	180 (79.3%)	47 (20.7%)
Do you think you overeat?	153 (67.4%)	74 (32.6%)
Do you think you eat inappropriate food?	126 (55.5%)	101 (44.5%)
Do you think you get enough exercise?	114 (50.2%)	113 (49.8%)
Do you think the chores of the house is exercise?	98 (43.2%)	129 (56.8%)
Have you ever taken Hakeemi medication?	57 (25.1%)	170 (74.9%)
Do you consider Hakeemi medications harmful?	45 (19.8%)	180 (80.2%)

## Discussion

Nearly 70% of all patients enrolled in the study were from urban centers; this is simply a representation of the diverse group of people treated at JPMC on a daily basis. All reports from urban centers about metS, type II diabetes mellitus, and NAFLD have previously illustrated a disproportionately raised prevalence and incidence rates among women [23]. However, the differences between the rates for men and women for all three entities were comparable and only became apparent in later age groups (i.e., 40 years or above) [24]. Three-quarters of our participants were female, but the higher incidence of NAFLD in women cannot explain this observation to this degree. Our opinion is that this matter reflects complex social circumstances and is therefore beyond the scope of this cohort. The mean age recorded in our study was 39.85 ± 9.79 years; this relatively young age is consistent with the obesity pandemic that is now affecting an ever younger populace and is being reported universally [4,14,23,24].

Providing objective parameters regarding a person’s socioeconomic status is nearly impossible in today’s volatile economic environment. Therefore, we did not define economic status per se; instead, this choice was made by the patients themselves. Traditionally, NAFLD has been associated with a high income and the lifestyle that accompanies it, but our results do not concur with this association [25]. Our observations verify that NAFLD has evolved over the years and is no longer affected by the economic status of the patients. Even in the most impoverished societies, a sedentary lifestyle and the obesity pandemic seem to have taken hold (this is more evident in Pakistani rural societies, where daily wages are as low as 1 USD per day). Moreover, as with other aspects of NAFLD, this phenomenon is now being observed and reported more frequently [4,26].

We surveyed five distinct aspects of NAFLD. Questions 1-3 asked whether the patients knew they were harboring NAFLD. The follow-up questions determined whether the patients actually knew or even had the slightest idea of what NAFLD was and what it entailed for their cardiovascular risk. Only one in four patients knew about being diagnosed with NAFLD, only half of those patients knew what NAFLD was, and even fewer were aware of their increased cardiovascular risk due to NAFLD. Overall, only one in nine patients was fully aware of one's disease. Several previous authors have mentioned the indifferent attitudes patients have toward NAFLD [20-22]. Nevertheless, the massive gap observed in our study between disease prevalence and the lack of NAFLD knowledge has not been reported in the current literature. To the best of our knowledge, this is the first study on the perception of NAFLD in Pakistan. Any comparison of data between developed and underdeveloped parts of the world is difficult due to the huge differences in standards of living and healthcare systems; however, with our evidence, we can safely assume that the pitfalls seen with respect to NAFLD and its perception in developed nations are further widened in states like Pakistan.

Obesity, unlike NAFLD, is easily observed even by a third party. Questions 4-6 analysed the perception of body weight among our participants. A mean BMI of 31.21 ± 3.6 kg/m^2^ verified that obesity was rampant among our subjects. Weight loss and, by extension, healthy eating/nutrition are the gold standards for managing NAFLD [27,28]. For this reason, the observation that 8 in 10 patients recognized the need for weight loss was heartening. Still, a small proportion did not accept being overweight, and those patients required extensive counseling. A high number of patients were willing to undergo weight-loss programs, but most did not admit to their unhealthy eating habits. About half admitted to frequent overeating, and approximately 44% admitted to eating unhealthy foods. Similar positions were seen pertaining to the need for more exercise/physical activity.

This denial of unhealthy eating habits and the lack of physical activity represent the greatest, but most amenable, hurdle in tackling the epidemic of NAFLD. Counseling through doctors, specialists, and dieticians can play an important role in reaching this goal, but systemic changes must be instituted to curb the disease. Encouraging exercise, providing healthier alternatives to fast foods and energy drinks, educating the youth, promoting sports, and convening advertising campaigns on the consumption of more fresh fruits and vegetables are just a few strategies that can be employed on a large scale to avert a healthcare disaster.

We also independently evaluated one factor that affects liver pathology, namely, “Hakeemi” medications. These are quack medications that are neither registered nor regulated by any authority but are regularly used in Pakistani society [29]. The constituents of these “drugs” are not known, side effects are not recorded, and dosages are not set. They can be administered by virtually anyone via any route, do not have labels, can be used (falsely) for any and all ailments, have no preparation details, and can be prescribed through word of mouth only (i.e., no written prescription), with no evidence of the transactions between the patients and quacks. These medications are also notorious for causing renal dysfunction [30]. Liver damage has been reported, but no definitive data are available locally, and only expert opinions or recommendations exist. Similarly, “Hakeemi” preparations have been reported to cause fatty liver, but again no definitive proof exists, for the reasons discussed above. The main point we wanted to highlight in the present study was just how comfortable the general populace is with the use of these deleterious substances. Only a quarter of the patients had previously used “Hakeemi” substances, yet more than 80% did not consider them harmful. This represents considerable confidence in products with such extreme shortcomings without any experience with them. The use of these substances represents a longstanding problem in the region, but it has been difficult to control. Since no drugs are specifically approved for NAFLD, quacks can lure patients with false promises of disease resolution, and this has been seen to cause further deterioration in other illnesses [30].

Limitations of the study

We did not differentiate between the different stages of NAFLD (or NASH) via biopsy or histopathology. Educational status was not included in the study to recruit a wider array of participants; however, this could have led to human bias and lack of understanding in some patients.

## Conclusions

Patients with NAFLD have little knowledge about the disease they harbor, its nature, its implications, and the need for intervention. This represents a major gap between disease causality and its understanding and eventual outcomes that can only be bridged by introducing policy in the form of clinical guidelines, awareness campaigns, civil society involvement, and healthcare system reforms including registries. For the moment, further exploration of the awareness of and attitudes towards NAFLD is needed to develop effective strategies for combating this disease in Pakistan.

## References

[1] Ludwig J, Viggiano TR, McGill DB, Oh BJ (1980). Nonalcoholic steatohepatitis: Mayo Clinic experiences with a hitherto unnamed disease. Mayo Clin Proc.

[2] Lindenmeyer CC, McCullough AJ (2018). The natural history of nonalcoholic fatty liver disease—an evolving view. Clin Liver Dis.

[3] Alexander M, Loomis AK, van der Lei J (2019). Risks and clinical predictors of cirrhosis and hepatocellular carcinoma diagnoses in adults with diagnosed NAFLD: real-world study of 18 million patients in four European cohorts. BMC Med.

[4] Younossi ZM, Koenig AB, Abdelatif D, Fazel Y, Henry L, Wymer M (2016). Global epidemiology of nonalcoholic fatty liver disease—meta-analytic assessment of prevalence, incidence, and outcomes. Hepatology.

[5] Altamirano J, Qi Q, Choudhry S (2020). Non-invasive diagnosis: non-alcoholic fatty liver disease and alcoholic liver disease. Transl Gastroenterol Hepatol.

[6] Allison DB, Downey M, Atkinson RL (2008). Obesity as a disease: a white paper on evidence and arguments commissioned by the Council of the Obesity Society. Obesity (Silver Spring).

[7] Gaissmaier W (2019). A cognitive-ecological perspective on risk perception and medical decision making. Med Decis Making.

[8] Rafal RD (1994). Neglect. Curr Opin Neurobiol.

[9] DeJong H, Hillcoat J, Perkins S, Grover M, Schmidt U (2012). Illness perception in bulimia nervosa. J Health Psychol.

[10] Bhagavathula AS, Aldhaleei WA, Rahmani J, Mahabadi MA, Bandari DK (2020). Knowledge and perceptions of COVID-19 among health care workers: cross-sectional study. JMIR Public Health Surveill.

[11] Cori L, Bianchi F, Cadum E, Anthonj C (2020). Risk perception and COVID-19. Int J Environ Res Public Health.

[12] Man MA, Toma C, Motoc NS (2020). Disease perception and coping with emotional distress during COVID-19 pandemic: a survey among medical staff. Int J Environ Res Public Health.

[13] Führer A, Frese T, Karch A (2020). COVID-19: knowledge, risk perception and strategies for handling the pandemic. (Article in German). Z Evid Fortbild Qual Gesundhwes.

[14] Cusi K (2016). Treatment of patients with type 2 diabetes and non-alcoholic fatty liver disease: current approaches and future directions. Diabetologia.

[15] Chen SH, He F, Zhou HL, Wu HR, Xia C, Li YM (2011). Relationship between nonalcoholic fatty liver disease and metabolic syndrome. J Dig Dis.

[16] Targher G, Tilg H, Byrne CD (2021). Non-alcoholic fatty liver disease: a multisystem disease requiring a multidisciplinary and holistic approach. Lancet Gastroenterol Hepatol.

[17] Alexander M, Loomis AK, van der Lei J (2019). Non-alcoholic fatty liver disease and risk of incident acute myocardial infarction and stroke: findings from matched cohort study of 18 million European adults. BMJ.

[18] Perumpail RB, Wong RJ, Ahmed A, Harrison SA (2015). Hepatocellular carcinoma in the setting of non-cirrhotic nonalcoholic fatty liver disease and the metabolic syndrome: US experience. Dig Dis Sci.

[19] Westfall E, Jeske R, Bader AR (2020). Nonalcoholic fatty liver disease: common questions and answers on diagnosis and management. Am Fam Physician.

[20] Goh GB, Kwan C, Lim SY (2016). Perceptions of non-alcoholic fatty liver disease - an Asian community-based study. Gastroenterol Rep (Oxf).

[21] de Silva HJ, Dassanayake AS (2009). Non-alcoholic fatty liver disease: confronting the global epidemic requires better awareness. J Gastroenterol Hepatol.

[22] Patel PJ, Banh X, Horsfall LU (2018). Underappreciation of non-alcoholic fatty liver disease by primary care clinicians: limited awareness of surrogate markers of fibrosis. Intern Med J.

[23] Wong MCS, Huang JLW, George J (2019). The changing epidemiology of liver diseases in the Asia-Pacific region. Nat Rev Gastroenterol Hepatol.

[24] Fan JG, Farrell GC (2009). Epidemiology of non-alcoholic fatty liver disease in China. J Hepatol.

[25] Mosca A, De Cosmi V, Parazzini F, Raponi M, Alisi A, Agostoni C, Nobili V (2019). The role of genetic predisposition, programing during fetal life, family conditions, and post-natal diet in the development of pediatric fatty liver disease. J Pediatr.

[26] Shiha G, Alswat K, Al Khatry M (2021). Nomenclature and definition of metabolic-associated fatty liver disease: a consensus from the Middle East and North Africa. Lancet Gastroenterol Hepatol.

[27] Vilar-Gomez E, Martinez-Perez Y, Calzadilla-Bertot L (2015). Weight loss through lifestyle modification significantly reduces features of nonalcoholic steatohepatitis. Gastroenterology.

[28] Ullah R, Rauf N, Nabi G, Ullah H, Shen Y, Zhou YD, Fu J (2019). Role of nutrition in the pathogenesis and prevention of non-alcoholic fatty liver disease: recent updates. Int J Biol Sci.

[29] Khan R, Mustufa MA, Hussain S (2020). Factors contributing to the public proneness towards quacks in Sindh. Pan Afr Med J.

[30] Jafri L, Khan AH, Asif H (2020). Kidney stone analysis and spiritual guides (pir faqir): the known-unknowns. J Pak Med Assoc.

